# Endoscopic Management of Infected Necrotizing Pancreatitis: an Evidence-Based Approach

**DOI:** 10.1007/s11938-018-0189-8

**Published:** 2018-07-20

**Authors:** Lotte Boxhoorn, Paul Fockens, Marc G. Besselink, Marco J. Bruno, Jeanin E. van Hooft, Robert C. Verdonk, Rogier P. Voermans, on behalf of the Dutch Pancreatitis Study Group

**Affiliations:** 1 Department of Gastroenterology & Hepatology, Amsterdam Gastroenterology and Metabolism, Amsterdam UMC, University of Amsterdam, Meibergdreef 9, 1105 AZ Amsterdam, the Netherlands; 2 Department of Surgery, Amsterdam Gastroenterology and Metabolism, Amsterdam UMC, University of Amsterdam, Meibergdreef 9, Amsterdam, 1105 AZ the Netherlands; 3 Department of Gastroenterology & Hepatology, Erasmus MC University Medical Center, Rotterdam, the Netherlands; 4 Department of Gastroenterology & Hepatology, St. Antonius Hospital, Nieuwegein, the Netherlands

**Keywords:** Endoscopic drainage, Endoscopic necrosectomy, Infected necrotizing pancreatitis, Walled-off necrosis, Lumen-apposing metal stent

## Abstract

**Purpose of Review:**

Endoscopic management of infected necrotizing pancreatitis has evolved rapidly over the past years and there have been interesting innovations in this field. This review provides an update on the most recently published literature regarding endoscopic management of infected necrotizing pancreatitis.

**Recent Findings:**

A recent randomized trial demonstrated no difference in mortality and major morbidity between endoscopic and surgical step-up treatment of infected necrotizing pancreatitis. However, endoscopic therapy resulted in shorter hospital stay and less pancreatic fistulas. Various innovations have been investigated with the aim to further optimize endoscopic therapy, in particular lumen-apposing metal stents. While major stent-related complications were also reported, findings from recent studies indicated that their use was associated with higher resolution rates of walled-off necrosis compared to double-pigtail stents. Other innovations, such as the multiple gateway technique and dual-modality mode, can be considered for treatment of particular cases. Furthermore, research suggests that irrigation of walled-off necrosis can be performed by using a nasocystic tube and discontinuation of proton-pump inhibitors may be considered.

**Summary:**

Endoscopic treatment should be the preferred treatment modality in patients with infected necrotizing pancreatitis who are eligible for endoscopic drainage. Although data suggests that lumen-apposing metal stents are superior to double-pigtail stents, prospective multicenter studies focusing on safety as well as long-term follow-up are first needed.

## Introduction

Acute pancreatitis is the most common gastrointestinal diagnosis requiring hospital admission [1]. Most patients experience a mild clinical course and can be managed with fluid resuscitation and pain control. Approximately 20% of patients develop a severe pancreatitis with necrosis of the (peri)pancreatic tissue [2].

In the majority of cases, (peri)pancreatic necrosis remains sterile and can be treated conservatively. However, about 30% of patients develop infected (peri)pancreatic necrosis [3]. This can be diagnosed by the presence of gas in the necrotic collection on abdominal imaging, a positive culture of a fine-needle aspiration (FNA) or clinical signs of infection (e.g., clinical deterioration despite supportive treatment, fever, increasing inflammatory markers, and/or positive blood cultures) [4].

Infected (peri)pancreatic necrosis is associated with high morbidity and mortality and requires intervention in the majority of cases [5, 6••]. Current guidelines advocate to delay intervention until the necrotic tissue has become walled-off (WON) [7]. It is important to underline that WON is a different entity than a pseudocyst. Especially in older literature, the term pseudocyst is used for WON as well as for real pseudocysts. Per definition, pseudocysts do not contain necrosis and therefore require a different approach. Definitions of pancreatic fluids collections (PFCs) are clearly described in the revised Atlanta classification [8].

The introduction of minimally invasive endoscopic, radiologic, and surgical modalities led to a considerable reduction in morbidity and mortality in comparison with traditional open surgery [9]. The first successful endoscopic drainage of WON was described in 1996 [10]. Subsequently, endoscopic ultrasound (EUS) was integrated, increasing technical success and the safety of the procedure [11, 12].

Endoscopic management of infected necrotizing pancreatitis has evolved rapidly ever since. This review provides an overview of most recently published evidence and innovations in this field.

### Evidence for endoscopic management of infected necrotizing pancreatitis

Over the last two decades, there has been a shift in the management of infected necrotizing pancreatitis towards less invasive approaches. Originally, primary open necrosectomy was performed at an early stage of disease, but was associated with high mortality rates [13]. In the past 10 years, minimally invasive surgical treatment within a “step-up” framework showed to reduce mortality and has replaced open surgery as the standard treatment [5].

The step-up approach can also be performed endoscopically. The findings from two randomized controlled trials by the Dutch Pancreatitis Study Group (DPSG) suggest that the endoscopic step-up approach is a potentially less invasive alternative to surgery.

The PENGUIN trial was the first randomized controlled trial (RCT) comparing endoscopic necrosectomy to surgical necrosectomy in 22 patients with infected WON [14]. Findings of the study demonstrated that endoscopic necrosectomy significantly reduced the inflammatory response and the occurrence of new-onset multiorgan failure (0 vs. 50%, *P* = 0.03).

Subsequently, the TENSION trial was initiated, which compared the endoscopic step-approach with the surgical step-up approach in patients with infected necrotizing pancreatitis [6••]. This trial showed no difference in mortality and major morbidity between both approaches (43% in endoscopic arm vs. 45% in surgical arm, *P* = 0.88). However, the endoscopic approach resulted in shorter hospital stay (mean 53 vs. 69 days, *P* = 0.014) and less pancreatic fistulas (5 vs. 32%, *P* = 0.0011). The endoscopic step-up approach was also superior to the surgical step-up approach in terms of (in)direct medical costs, which was likely due to the shorter length of hospital stay for endoscopically treated patients.

In conclusion, these findings suggest that endoscopic treatment should be the preferred treatment modality in patients for whom the infected WON can be reached transluminally.

### Recent innovations in endoscopic drainage of infected necrotizing pancreatitis

Endoscopic management of infected necrotizing pancreatitis has evolved rapidly over the past years and there have been interesting innovations in this field (Table 1).

**Table 1:** Recent innovations regarding endoscopic management of WON

	Innovation	Available evidence	Level of evidence*
Endoscopic drainage	• Lumen-apposing metal stents	Systematic review of case-controlled studies/cohort studies	2a-3a
	• Multiple transluminal gateway technique	Case series	4
	• Dual-modality mode	Case series	4
Endoscopic necrosectomy	• Hydrogen peroxide	Case series Prospective open label study	4
	• Nasocystic irrigation	Case series	4
	• Discontinuation of proton-pump inhibitors	Case series	4
	• Endoscopic vacuum-assisted closure system	Case reports	4

*Grading according to the Oxford Levels of Evidence

#### Metal stents

Metal stents were proposed as an alternative to the traditionally used double-pigtail stents (DPS) for endoscopic drainage of WON. DPS are limited by a small lumen (7 French) that may result in inadequate drainage. A larger diameter of metal stents would permit spontaneous passage of necrotic tissue into the stomach, improving the success rate of endoscopic drainage.

Although originally designed for biliary strictures, fully covered self-expandable metal stents (FC-SEMS) were the first metal stents assigned for endoscopic drainage. Several case series have described success rates ranging between 80 and 100% when FC-SEMS were used for the endoscopic drainage of WON [15–18]. However, high rates of stent migration were also reported (15%) [19].

**Fig. 1:**
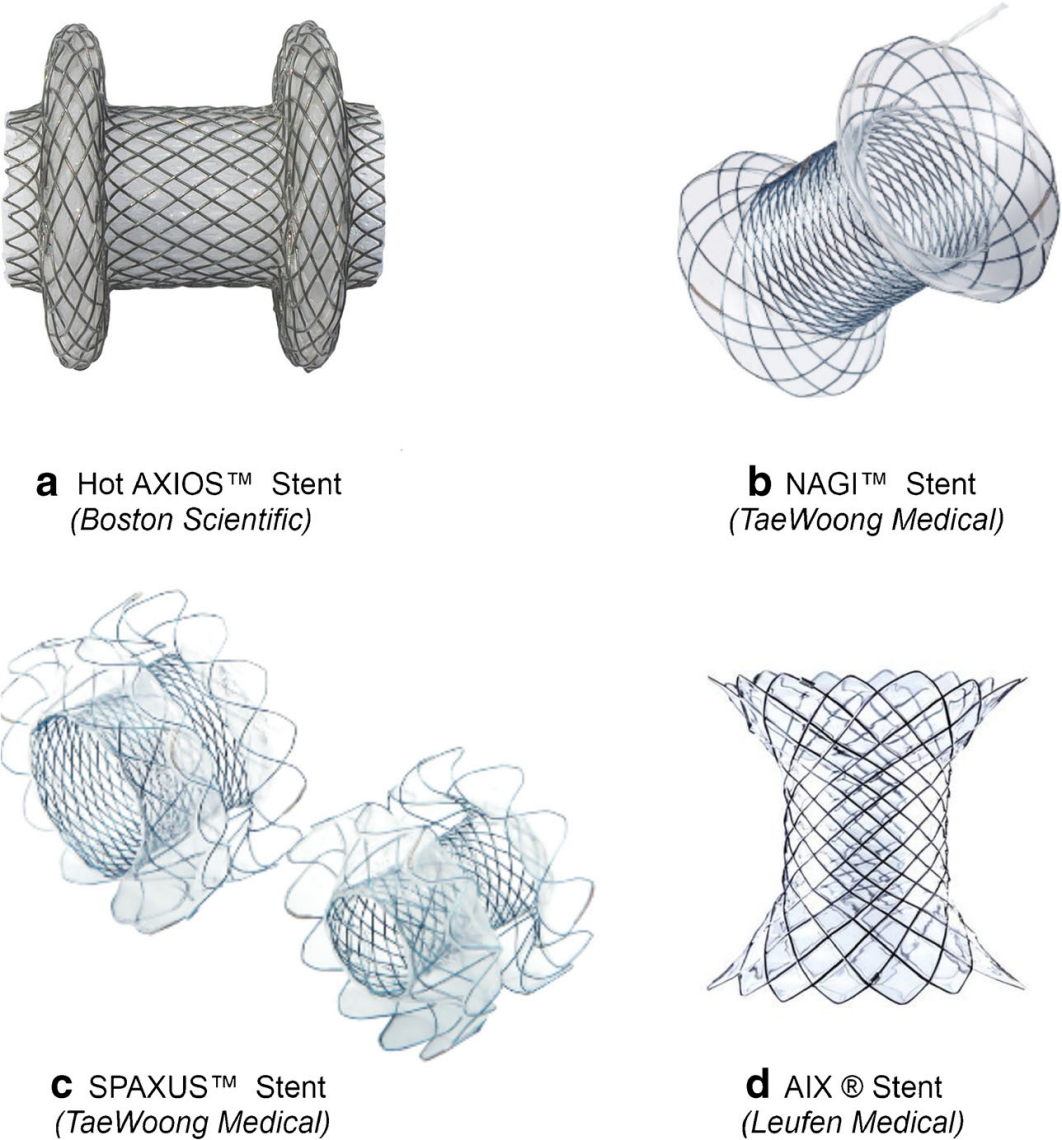
Overview of the available lumen-apposing metal stents

Lumen-apposing metal stents (LAMS) were designed in order to decrease the risk of stent migration. The stents have a saddle-shaped design with anchoring flanges and were originally applied to prevent leakage between the walls of both collections and the stomach (Fig. 1). Its large diameter also permits the spontaneous passage of necrotic tissue into the stomach, which may reduce the need for necrosectomy. LAMS also provide, if necessary, an easy entry port for endoscopic necrosectomy.

Over 30 retrospective case series, four prospective cohort studies and one interim analysis of a RCT evaluated the efficacy of LAMS for drainage of PFCs (Table 2). A recent review evaluated the endoscopic drainage of WON using DPS versus metal stents from published studies between 1990 and 2016 [20••]. The authors included 41studies, mainly retrospective and non-comparative studies, regarding the use of DPS and metal stents (total of 2213 patients). Only five of the studies included in the meta-analysis compared DPS directly to metal stents (note that the authors excluded four studies due to low total number of patients and disproportion of DPS versus metal stents) [21••, 22•, 23–25]. Overall, resolution of WON was more likely to occur when using LAMS (91.5%) compared to DPS (80.9%) (OR 2.5, 95% CI 1.4–4.3, *P* = 0.001). No difference was found in the resolution of WON after the initial endoscopic procedure with LAMS (52.3%) compared to DPS (43.4%) (OR 1.4, 95% CI 0.56–3.6, *P* = 0.4).

**Table 2:** Prospective studies about the use of LAMS in the management of WON

Author	Study design	WON (*N*)	LAMS	Stent removal	Follow-up	Adverse events
Dhir et al. *(2017)*	Prospective study Single center	88	NAGI™ stent	Median 3.5 weeks (range 3–17 weeks)	Median 22 months (range 3–46 months)	Mild* • Post-ERCP pancreatitis following ERCP and ductal stenting (*N* = 3) • Stent migration (*N* = 2) Moderate* • Bleeding (*N* = 1) Severe* • Bleeding (*N* = 2) • Abscess that required surgery (*N* = 2) Fatal* • Death (cause unknown) (*N* = 1)
Bang et al. (2016)	Randomized controlled trial Single center	21	Hot AXIOS™ stent (*ø* 15 mm)	(Ongoing trial)	(Ongoing trial)	• Bleeding (*N* = 3) • Buried LAMS syndrome (*N* = 2) • Biliary stricture (*N* = 1)
Gornals et al. (2016)	Prospective study Single center	13	AXIOS™/Hot AXIOS™ stent (*ø* 10 or 15 mm)	Mean 9 ± 3.4 weeks (range 4–16)	Mean 13.3 ± 11.4 months (range 2–36)	Moderate* • Migration/infection (*N* = 1)^a^ • Occlusion/infection (*N* = 1)^b^ Severe* • Bleeding (*N* = 2)
Walter et al. (2015)	Prospective study Multicenter	46	AXIOS™ stent (*ø* 10 or 15 mm)	Median 32 days (range 2–178)	NR	• Infection/occlusion (*N* = 4) • Perforation (*N* = 1)
Shah et al. (2015)	Prospective study Multicenter	11	AXIOS™ stent (*ø* 10 or 15 mm)	NR	NR	• Partial occlusion resulting in stent removal (*N* = 1) • Dislodgement during DEN resulting in precut and surgical debridement (*N* = 1) • Fever with prolonged hospitalization (*N* = 1) • Abdominal pain requiring endoscopy (*N* = 1)

*DEN* direct endoscopic necrosectomy, *ERCP* endoscopic retrograde cholangio-pancreatography, *LAMS* lumen-apposing metal stents

*Grading according to the American Society for Gastrointestinal Endoscopy Lexicon criteria

^a^ Treated with antibiotics and placement of a new LAMS

^b^ Treated with antibiotics and placement of a double pigtail through the AXIOS stent

To date, there are conflicting literature reports with respect to the safety of LAMS in WON drainage. The aforementioned meta-analytical review indicated that the use of LAMS (in the non-comparable studies) was associated with lower bleeding (6.2 vs. 12.6%, *P* = 0.007) and lower stent occlusion (7.5 versus 17.4%, *P* = 0.015) [20••]. However, the results of an ongoing randomized controlled trial about endoscopic drainage of WON with LAMS versus DPS have raised concerns [21••]. Stent-related adverse events were observed in 6 of the 12 patients randomized to LAMS: major bleeding (*N* = 3), buried stent syndrome (*N* = 2), and biliary stricture (*N* = 1) were reported [21••]. Interestingly, all adverse events occurred within 6 weeks after stent placement. As a result, the authors changed the study protocol by performing imaging 3 weeks after stent placement in order to remove the LAMS as soon as the necrotic collection was collapsed. These findings suggest that the risk of stent-related adverse events may be related to the timing of LAMS removal. Yet, current literature provides no consensus about the appropriate timing of LAMS retrieval: an interval ranging from 3.5 to 10 weeks after the initial drainage procedure has been described [21••, 26–28]. Currently, the ESGE guidelines recommend to remove the LAMS after a maximum of 4 weeks [29••]. In order to minimize the risks of bleeding and migration, it has also been suggested to place an additional DPS through the lumen of the LAMS. A single-center retrospective was conducted to compare LAMS alone (*N* = 21) versus the combination of LAMS and DPS (*N* = 20) and showed that adverse events were significantly higher in the LAMS group compared to the LAMS combined with DPS group (43 versus 10%, *P* = 0.04) [30].

Endoscopic drainage with LAMS may be restricted by its high costs. To date, high-quality methodologic studies comparing the cost-effectiveness of the different stents used for endoscopic drainage are lacking. In a recent study from the USA, the costs of endoscopic drainage with LAMS per patient were higher compared to DPS ($20,029 vs. $15,941) [31]. However, more patients achieved successful endoscopic drainage of WON with LAMS (92 vs. 84%). For this reason, the authors described that the incremental cost-effectiveness ratio favored drainage with LAMS.

In contrast, the theoretical benefit of LAMS is not present in the endoscopic drainage of pancreatic pseudocysts. A meta-analysis showed no difference in decrease in PFC size and/or resolution of symptoms between patients with pseudocysts treated with DPS (85%, 95% CI 81–89%) or with metal stents (83%, 95% CI 74–89%) [32]. Although randomized trials are lacking, DPS should be preferred over LAMS when draining pseudocysts given their excellent safety profile and the possibility to leave DPS in for longer periods of time.

To summarize, endoscopic drainage with LAMS seems to result in higher resolution rates of WON when compared with DPS. Therefore, LAMS should be considered in patients with infected WON who are eligible for endoscopic drainage. Nonetheless, the best available evidence originates from only a few prospective studies. Moreover, important questions regarding the safety of LAMS are still unanswered. The current recommendation to remove LAMS after a maximum of 4 weeks is also not based on high-quality evidence [29••]. The suggestion that the placement of DPS within LAMS would minimize the risk of adverse events should be further looked into, although it theoretically conflicts with the proposed drainage advantages associated with the large diameter of LAMS. If a necrotic collection is not fully collapsed, LAMS should be exchanged for DPS, but this may not be easy or even possible in all patients. Finally, it is unclear whether LAMS are cost-effective. In conclusion, these concerns need to be addressed by future multicenter prospective studies before LAMS can be advised for routine use.

We have recently initiated a prospective multicenter clinical study investigating the use of LAMS (Hot AXIOS™) in the management of infected WON (AXIOMA study, NTR7056). With this study, we aim to evaluate the treatment outcomes and safety of LAMS. Furthermore, AXIOMA will test the hypothesis that the use of LAMS is more cost-effective by reducing the need of additional necrosectomies.

#### Other approaches to optimize endoscopic drainage

Besides the administration of large diameter metal stents, optimization of endoscopic drainage may be achieved by the creation of multiple transluminal tracts into the WON cavity, also called the “multiple transluminal gateway technique” (MTGT).

Three retrospective case series compared MTGT to conventional drainage techniques for the treatment of WON [33–35]. In total, 39 of 204 patients (19%) were treated with MTGT. MTGT was applied in patients who responded inadequately to the initial endoscopic drainage procedure and in case WON exceeded ≥ 12 cm [33, 35]. A maximum of three transluminal tracts were created. Treatment success between 92 and 100% was reported in patients receiving MTGT compared to 52–70% receiving conventional drainage techniques. One disadvantage of MTGT was a significantly longer median procedural duration in comparison with the conventional drainage technique (37 vs. 22 min, *P* = 0.017) [35]. However, the introduction of a LAMS allowed a more rapid creation of multiple transluminal tracts (< 10 min) [36].

Transluminal drainage can be combined with percutaneous drainage in particular cases of WON extending to the paracolic gutters. This is also referred to as “dual-modality drainage” (DMD).

Five retrospective studies, which were all initiated in the same hospital, reported on the DMD technique [37–41]. One of the studies was comparative and described 49 patients that completed DMD versus 45 patients that were only drained percutaneously [37]. It showed that the DMD technique was associated with a shorter length of hospitalization compared to percutaneously draining only (mean 24 vs. 54 days, *P* < 0.002). Additionally, DMD-treated patients required less drains (1.3 vs. 1.9, *P* < 0.001). Lastly, a lower number of endoscopic procedures (1.9 vs. 2.7, *P* < 0.02) and CT scans (7.8 vs. 14.0, *P* < 0.001) were needed. In another retrospective study, a total of 117 patients underwent DMD for either infected WON (*N* = 55) or symptomatic sterile WON (*N* = 62) [38]. Interestingly, none of the 103 patients that completed treatment (88%) needed an additional (surgical) necrosectomy. Furthermore, not a single patient developed a pancreaticocutaneous fistula after treatment with the DMD technique.

In conclusion, evidence suggests that MTGT and DMD are potentially effective approaches for the optimization of endoscopic drainage of WON. Although, these findings were obtained in retrospective studies only and further prospective studies are needed. Therefore, MTGT should only be considered in selected cases of patients who not respond well on the initial endoscopic drainage procedure or in the case of in large WON. Additional percutaneous drainage can be initiated if WON extends to the paracolic gutters.

### Recent innovations in endoscopic necrosectomy techniques

Endoscopic necrosectomy remains to be a challenging and time-consuming procedure, especially due to the lack of dedicated endoscopic devices and a standardized approach. Yet, several innovations have been explored in order to simplify the debridement of necrotic tissue.

#### Hydrogen peroxide

One of the reported innovations is the use of hydrogen peroxide (H_2_O_2_) for the irrigation of WON. H_2_O_2_ decomposes into water and oxygen when combining with organic tissue and is therefore thought to facilitate the removal of necrotic debris.

Four retrospective studies and one prospective open label study reported on the use of H_2_O_2_ for the debridement of WON [42–46]. A total of 108 patients underwent endoscopic drainage of WON followed by H_2_O_2_ irrigation. Drainage was performed with DPS, FC-SEMS, and LAMS. The reported number of required necrosectomy procedures ranged from 1 to 3. Resolution of WON occurred in 79 to 100% of patients.

Based on this limited set of data, there is currently no supportive evidence to recommend H_2_O_2_ in the treatment of WON. Moreover, its safety is questionable because several case series reported that its use may be related to embolic events [47–52]. In conclusion, irrigation with H_2_O_2_ is currently not advised.

#### Nasocystic irrigation

Irrigation of WON can also be performed by the placement of a nasocystic tube. The tube can be inserted next to DPS or through a deployed metal stent.

To date, prospective studies assessing the duration, type, or volume of irrigation with a nasocystic tube are lacking. In a retrospective comparative study, patients treated with a nasocystic tube reported a decrease in symptoms after 1 month in combination with a minimum of 30% decrease in WON size on imaging (85 vs. 63%, *P* = 0.03). Furthermore, the addition of a nasocystic tube to placed DPS decreased stent occlusion rates as compared to using the stent alone (13 vs. 33%, *P* = 0.03) [53].

It is not clear whether irrigation with a nasocystic tube offers any advantages when combined with a LAMS. In a large multicenter retrospective study, 22 of 68 patients with WON were drained via a LAMS with the addition of a nasocystic tube. In this study, there was not a difference in WON resolution in patients drained with or without nasocystic tube (90.9 vs. 95.6%, *P* = 0.59) [54].

Although data on the irrigation with a nasocystic tube is scarce, its use can be recommended when a significant amount of necrosis is present in the necrotic collection.

#### Discontinuation of proton-pump inhibitors

The role of gastric acid in the chemical debridement of necrotic tissue has been investigated as well. It is thought that the low pH of gastric acid facilitates the liquefaction of necrosis and prevents bacterial overgrowth. Because proton-pump inhibitors (PPIs) cause a long-lasting reduction of stomach acid production, they could have negative effects on the resolution of WON.

So far, only two studies have focused on the role of PPI and WON resolution [55, 56]. A retrospective multicenter study investigating patients with WON showed that more necrosectomy procedures were required to achieve WON resolution in patients using PPIs (*N* = 136) in comparison with those (*N* = 136) who did not use PPIs (median number of procedures of 4.6 vs. 3.2, respectively) [56]. Another retrospective study of 71 patients (54 PPI users and 17 non PPI users) reported a similar trend (mean endoscopic procedures 2 vs. 1.4) [55].

Although this limited set of studies is insufficient to strongly recommend the discontinuation of PPIs in all endoscopically treated patients with WON, this can be considered in particular cases in which there is no strong indication to continue their use.

#### Endoscopic vacuum-assisted closure system

Vacuum-assisted closure (VAC) is an established treatment method for wounds. The negative pressure of a vacuum-sealed sponge results via several mechanisms into wound closure. Endoscopic vacuum-assisted closure (EVAC) is increasingly being conducted as a new method to repair upper gastrointestinal defects [57]. EVAC might be an effective addition to endoscopic necrosectomy if established endoscopic treatment options have failed.

However, only four case reports described the use of a transluminally placed vacuum-assisted closure system in the management of WON [58–61]. Moreover, its potential benefits may be limited because the system should often be exchanged, resulting in an added number of endoscopic interventions. Consequently, it is unsure whether EVAC will offer any advantages for the management of WON. Future studies should determine the efficacy of EVAC and address safety endpoints such as the risk of a pancreatic or intestinal fistula.

### The role of endoscopy in disconnected pancreatic duct syndrome

Disconnected pancreatic duct syndrome (DPDS) is an important but often overlooked complication in patients with acute necrotizing pancreatitis. Disruption of the main pancreatic duct due to necrosis may cause extraductal leakage of pancreatic fluid, resulting pancreatic fistulas, pseudocysts, ascites, and pleural effusion [62].

Several modalities, such as endoscopic retrograde cholangiopancreatography (ERCP), contrast-enhanced computed tomography (CECT), and magnetic resonance cholangiopancreatography (MRCP), have been conducted in order to diagnose DPDS [63, 64]. To date, it remains unclear which modality visualizes a disrupted pancreatic duct most accurately and should be preferred. The role of EUS was researched in a recent prospective observational study [65]. EUS was used to identify duct disruption in 21 patients during the initial EUS-guided drainage of WON. In all patients, EUS demonstrated the termination of the upstream pancreatic parenchyma and duct into the necrotic collection, thereby suggesting the presence of a duct disruption. These findings were afterwards confirmed by follow-up CECT (100%), ERCP (81%), EUS-pancreatogram (14%), and surgical pathology (5%).

Pancreatic duct disruption and associated fluid collections can be treated surgically, percutaneously, or endoscopically. Currently, endoscopic therapy has become increasingly important in the management of DPSD, especially when a PFC is present.

The current accepted endoscopic strategy for DPDS and associated PFCs (both WON and pseudocysts) is to perform endoscopic drainage with long-term indwelling DPS [29••]. A randomized controlled trial that showed that recurrence of PFCs was less likely if the transmural stents were left in situ (0 vs. 38%, *P* = 0.013) [66]. Another more recent retrospective study also suggested that recurrence of fluid collections was significantly lower in patients with long-term indwelling transmural stents (0 vs. 21%, *P* = 0.02) [33].

In contrast, literature is divergent regarding the benefits of transpapillary drainage of DPDS. Theoretically, stenting of the PD across the site of the disruption may direct the pancreatic flow preferentially into the duodenum [67]. A meta-analysis showed that the combination of transmural drainage and transpapillary drainage did not have additional benefit in recurrence of PFCs (OR 1.49, 95% CI 0.53–4.21, *P* = 0.45) or complications (OR 1.15, 95% 0.61–2.18, *P* = 0.67) [68]. Thus, combining transluminal drainage with routine stenting of the pancreatic duct is not recommended [29••].

In conclusion, DPDS is a serious complication of acute necrotizing pancreatitis and is characterized by the discontinuity of the main pancreatic duct. Long-term indwelling of transluminal DPS is recommended after transluminal WON drainage in case of a proven disconnected pancreatic duct [29••]. Consequently, metal stents should be replaced by DPS in patients with DPDS after approximately 4 weeks. EUS findings of Bang et al. suggest that a disrupted duct may be recognized at the time of initial EUS-guided drainage of WON [65]. In that case, endoscopists should consider transluminal drainage with DPS instead of a metal stent. The combination of transluminal drainage with routine stenting of the pancreatic duct is not recommended [29••].

## Conclusion

Over the past decade, endoscopic management of infected necrotizing pancreatitis has evolved rapidly. A recent randomized trial demonstrated no difference in mortality and major morbidity between endoscopic and surgical management step-up approach for infected necrotizing pancreatitis [6••]. Nonetheless, endoscopic therapy resulted in shorter hospital stay and less pancreatic fistulas and should therefore be the preferred treatment modality.

Secondly, there is some evidence that the use of LAMS is superior to the use of DPS for endoscopic drainage of WON. Yet, this needs to be confirmed by multicenter prospective studies, considering the reported safety issues on LAMS. Currently, it is recommended to retrieve LAMS after a maximum of 4 weeks in order to prevent stent-related complications [29••]. In case of a clear disconnected pancreatic duct, DPS should be preferred over LAMS, because DPS can be left in situ permanently. The use of LAMS is also not recommended for the drainage of pancreatic pseudocysts.

MTGT should be considered in patients who do not response well on initial drainage or in case of large WON. If WON extends to the paracolic gutter, the DMD approach can be initiated. Research suggests that irrigation of WON can be performed by using a nasocystic tube and the discontinuation of proton-pump inhibitors should be taken into consideration. To date, there is insufficient evidence that H_2_O_2_ and EVAC should be applied in the endoscopic management of WON.

## Abbreviations

CECT: Contrast-enhanced computed tomography
DEN: Direct endoscopic necrosectomy
DMD: Dual-modality drainage
DPDS: Disconnected pancreatic duct syndrome
DPS: Double-pigtail stents
DPSG: Dutch Pancreatitis Study Group
ERCP: Endoscopic retrograde cholangiopancreatography
EUS: Endoscopic ultrasound
FC-SEMS: Fully covered self-expandable metal stents
FNA: Fine-needle aspiration
H_2_O_2_: Hydrogen peroxide
LAMS: Lumen-apposing metal stents
MTGT: Multiple gateway technique
MRCP: Magnetic resonance cholangiopancreatography
PFCs: Pancreatic fluid collections
PPIs: Proton-pump inhibitors
RCT: Randomized controlled trial
VAC: Vacuum-assisted closure system
WON: Walled-off necrosis
